# Clinical Characteristics of Patients With Benign Paroxysmal Positional Vertigo Diagnosed Based on the Diagnostic Criteria of the Bárány Society

**DOI:** 10.3389/fneur.2020.00602

**Published:** 2020-07-03

**Authors:** Xia Ling, Dan-Hua Zhao, Bo Shen, Li-Hong Si, Kang-Zhi Li, Yuan Hong, Zhe-Yuan Li, Xu Yang

**Affiliations:** ^1^Department of Neurology, Aerospace Center Hospital, Peking University Aerospace School of Clinical Medicine, Beijing, China; ^2^Department of Neurology, Peking University International Hospital, Beijing, China; ^3^Department of Neurology, The Second Affiliated Hospital of Zhengzhou University, Zhengzhou, China; ^4^Department of Neurology, Peking University Shougang Hospital, Beijing, China

**Keywords:** benign paroxysmal positional vertigo, Bárány society, diagnostic criteria, possible BPPV, clinical characteristics, diagnostic strategies

## Abstract

**Objectives:** To analyze the clinical characteristics of patients with benign paroxysmal positional vertigo (BPPV) diagnosed based on the diagnostic criteria of Bárány Society, verify the clinical application value of the diagnostic criteria, and further explore the clinical problems associated with the diagnosis of possible BPPV.

**Methods:** A total of 481 patients with BPPV who were admitted from March 2016 to February 2019 were included. All patients were diagnosed by the Dix-Hallpike, straight head hanging and supine roll tests, the nystagmus was recorded using videonystagmography. For patients with possible BPPV (uncertain diagnosis), particle repositioning therapy and follow-up diagnosis were used to further clarify diagnosis.

**Results:** Based on Bárány Society's diagnostic criteria for BPPV, the distribution characteristics of different BPPV types were as follows: 159 (33.1%) patients had posterior canal BPPV-canalolithiasis (PC-BPPV-ca), 70 (14.6%) patients had horizontal canal BPPV-ca (HC-BPPV-ca), 55 (11.4%) patients had spontaneously resolved-probable-BPPV (Pro-BPPV), and 53 (11.0%) patients had HC-BPPV-cupulolithiasis (HC-BPPV-cu). In emerging and controversial BPPV, 51 (10.6%) patients had multiple canal BPPV (MC-BPPV), 30 (6.2%) patients had PC-BPPV-cu, and 19 (4.0%) patients had anterior canal BPPV-ca (AC-BPPV-ca), 44 (9.1%) patients had possible-BPPV (Pos-BPPV). Among the 44 patients with Pos-BPPV, 23 patients showed dizziness/vertigo without nystagmus during the initial positional test, five patients were possible MC-BPPV, four patients had persistent geotropic positional nystagmus lasting > 1 min when lying on both sides, and were considered to have Pos-HC-BPPV, four patients showed apogeotropic nystagmus when lying on one side, and were considered to have possible short-arm HC-BPPV, four patients showed geotropic nystagmus when lying on one side, and were considered to have Pos-HC-BPPV, three patients had down-beating nystagmus, lasing > 1 min, were considered to have Pos-AC-BPPV-cu. One patient showed transient apogeotropic positional nystagmus on both sides during the supine roll test, and was diagnosed with possible anterior arm HC-BPPV.

**Conclusions:** PC-BPPV-ca is the most common among patients with BPPV, followed by HC-BPPV-ca. In emerging and controversial BPPV, MC-BPPV, and Pos-BPPV were more common. For the diagnosis of Pos-BPPV, a combination of the history of typical BPPV, particle repositioning therapy and follow-up outcome is helpful to clarify the diagnosis.

## Introduction

Benign paroxysmal positional vertigo (BPPV) is the most common paroxysmal vestibular disorder, with a lifetime prevalence of 3–10% (1). BPPV is characterized by positional vertigo and nystagmus that is triggered by changing head position, i.e., otoliths that detached from utricle are dropped into the canal and move along with gravity when head position changes (2). According to anatomical structures, BPPV can be divided into posterior canal BPPV (PC-BPPV), horizontal canal BPPV (HC-BPPV), anterior canal BPPV (AC-BPPV), and multiple canal BPPV (MC-BPPV) (3). PC-BPPV and HC-BPPV are more prevalent, and AC-BPPV is very rare (4). According to the pathophysiology of BPPV, it can be divided into canalolithiasis (ca) and cupulolithiasis (cu). In 2015, experts from various countries in the International Bárány Society discussed and formulated the consensus diagnostic criteria for BPPV, which objectively reflects the status of diagnosis and treatment of BPPV in clinical practice (5). In addition to the common types of BPPV, emerging and controversial BPPV are also included in the diagnostic criteria. The diagnostic criteria interpret these BPPV types, and provide clinicians with criteria and information for diagnosing BPPV. Further validation of the diagnostic criteria, especially the emerging and controversial BPPV among Chinese population has important clinical significance.

Given the above background, we included 481 patients with BPPV who were admitted to our hospital, aimed to analyze the clinical distribution characteristics of different BPPV types based on Bárány Society's diagnostic criteria for BPPV, and further analyzed the problems associated with the diagnosis of possible BPPV.

## Subjects and Methods

### Subjects

A total of 481 patients with BPPV who were admitted to the Department of Neurology, Aerospace Center Hospital, Peking University Aerospace School of Clinical Medicine from March 2016 to April 2019 were included. All patients gave written informed consent. This study has been approved by the ethics committee of our hospital. All patients underwent routine neuro-otological examinations, such as cranial nerve examination, Romberg's test, and Fukuda test, pure tone audiometry, eye movement test, bithermal caloric test and dynamic position test. Eye movement test include gaze, saccade, smooth pursuit, and optokinetic nystagmus tests. Dynamic position tests include Dix-Hallpike, supine roll, and straight head hanging (SHH) tests. A videonystagmograph (VNG, Interacoustics, Denmark) was used to record the nystagmus in BPPV patients. If necessary, MRI and other examinations were performed to exclude central paroxysmal positional vertigo.

### Diagnostic Methods

The diagnosis of BPPV is mainly based on the diagnostic criteria for BPPV formulated by the Bárány Society in 2015 (5). BPPV types include PC-BPPV-ca, HC-BPPV-ca, HC-BPPV-cu, and spontaneously resolved-probable BPPV (Pro-BPPV), AC-BPPV-ca, PC-BPPV-cu, MC-BPPV, and possible-BPPV (Pos-BPPV). The latter four types are emerging and controversial types of BPPV.

The spontaneous nystagmus was recorded in a sitting position with or without visual fixation. Dix-Hallpike and supine roll and SHH tests were performed to induce nystagmus in BPPV patients, and then BPPV patients were classified and diagnosed according to the medical history and characteristics of nystagmus.

Diagnosis of the involved semicircular canal: (1) PC-BPPV was diagnosed if vertical upbeat nystagmus with or without torsional component was induced by Dix-Hallpike test, and the reversal of the nystagmus often occurred when returning to an upright position; if vertical upbeat nystagmus with torsional component was induced, the torsional component involved the beating of the upper pole of the eyes toward the affected side. (2) HC-BPPV was diagnosed if geotropic horizontal nystagmus was induced by supine roll test, and the side with stronger nystagmus was the affected side; if apogeotropic horizontal nystagmus was induced, the side with weaker nystagmus was the affected side. (3) AC-BPPV was diagnosed if vertical downbeat nystagmus with/without torsional component was induced by Dix-Hallpike test; if the vertical downbeat nystagmus with torsional component was induced, the torsion of the upper pole of the eyes was toward the affected side. (4) The diagnosis of MC-BPPV was based on the presence of the typical nystagmus of multiple canals involved on Dix-Hallpike, SHH, and supine roll tests. (5) Pro-BPPV was diagnosed if patients had a history of recurrent episodes of positional vertigo or dizziness, that can be induced when the patients changed from an upright position to a supine position or when patients were in a supine position and then rolled onto one side, and those recurrent episodes lasted less than 1 min. Nystagmus and vertigo were not observed in the position test, and the recurrent episodes were not attributable to other diseases (5).

Diagnosis of Pos-BPPV: (a) Dix-Hallpike, supine roll, and SHH tests only induced vertigo, but did not induce nystagmus, or induced atypical nystagmus disappeared after treatment with particle repositioning maneuvers; (b) multiple canal involvement were suspected, but the affected canals cannot be determined; (c) co-existence of peripheral and central positional nystagmus; (d) symptoms were not attributed to other diseases (5).

**Figure 1:**
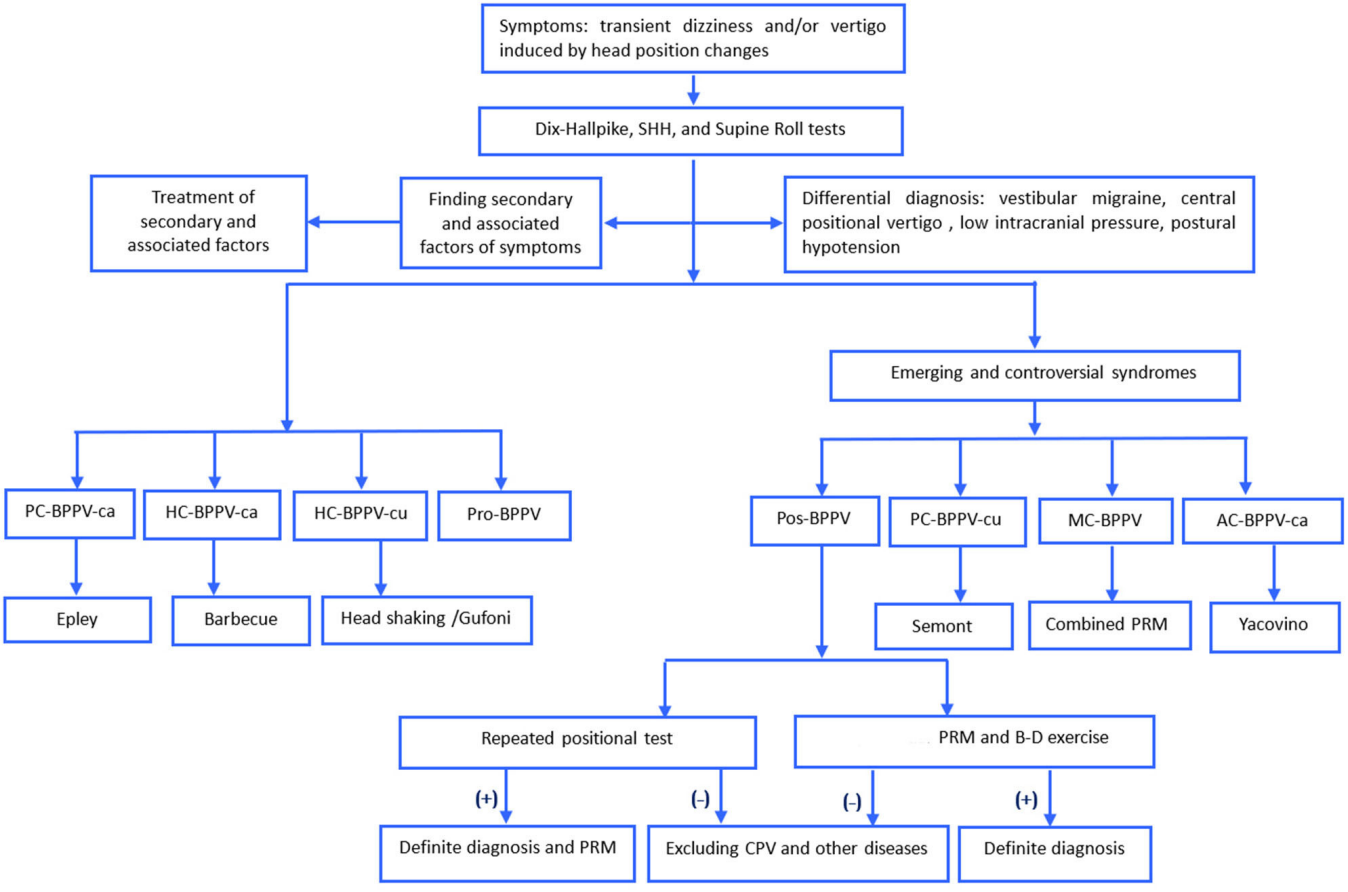
The process for diagnosis and treatment of BPPV. AC, anterior canal, ca, canalolithiasis; cu, cupulolithiasis; B-D, Brandt-Daroff; BPPV, benign paroxysmal positional vertigo, HC, horizontal canal, MC, multiple canal, PC, posterior canal, Pos, possible, PRM, particle repositioning maneuvers; Pro, probably spontaneously resolved, SHH, straight head hanging.

The detailed process for diagnosis and treatment of Pos-BPPV was shown in Figure 1. For patients with Pos-BPPV, the history of BPPV, particle repositioning therapy, and follow-up diagnosis were used to further confirm the diagnosis. (1) particle repositioning therapy was applied based on the type of vertigo and nystagmus characteristics induced by the position test and the affected side. Patients with Pos-PC-BPPV, -HC-BPPV, -AC-BPPV were treated with Epley, Barbecue, and Yacovino maneuvers, respectively. Patients who had much more complex nystagmus were treated with the combined particle repositioning therapy and/or Brandt-Daroff exercises. All patients with Pos-BPPV are followed up for one week and one month after treatment. (2) cupulolithiasis was considered if the duration of nystagmus was ≥1 min, and canalolithiasis was considered if the duration of nystagmus was <1 min.

After treatment with particle repositioning maneuvers, the Dix-Hallpike and supine roll tests were performed again at the same day or the next day. If all the nystagmus and vertigo disappeared, then a complete cure was achieved. Particle repositioning therapy was also considered effective if the nystagmus and vertigo was weaker without completely disappearing, whereas it was deemed to be non-effective if the nystagmus was unchanged or became further aggravated (6).

## Results

Among the 481 patients with BPPV included in this study, 157 (32.6%) patients were males, and 324 (67.4%) were females; the male to female ratio was 1:2.06. The average age of patients was 59.6 ± 14.9 years (range: 20–90 years), and the peak age of onset was 51–70 years, accounting for 52.4% of all patients with BPPV.

**Figure 2:**
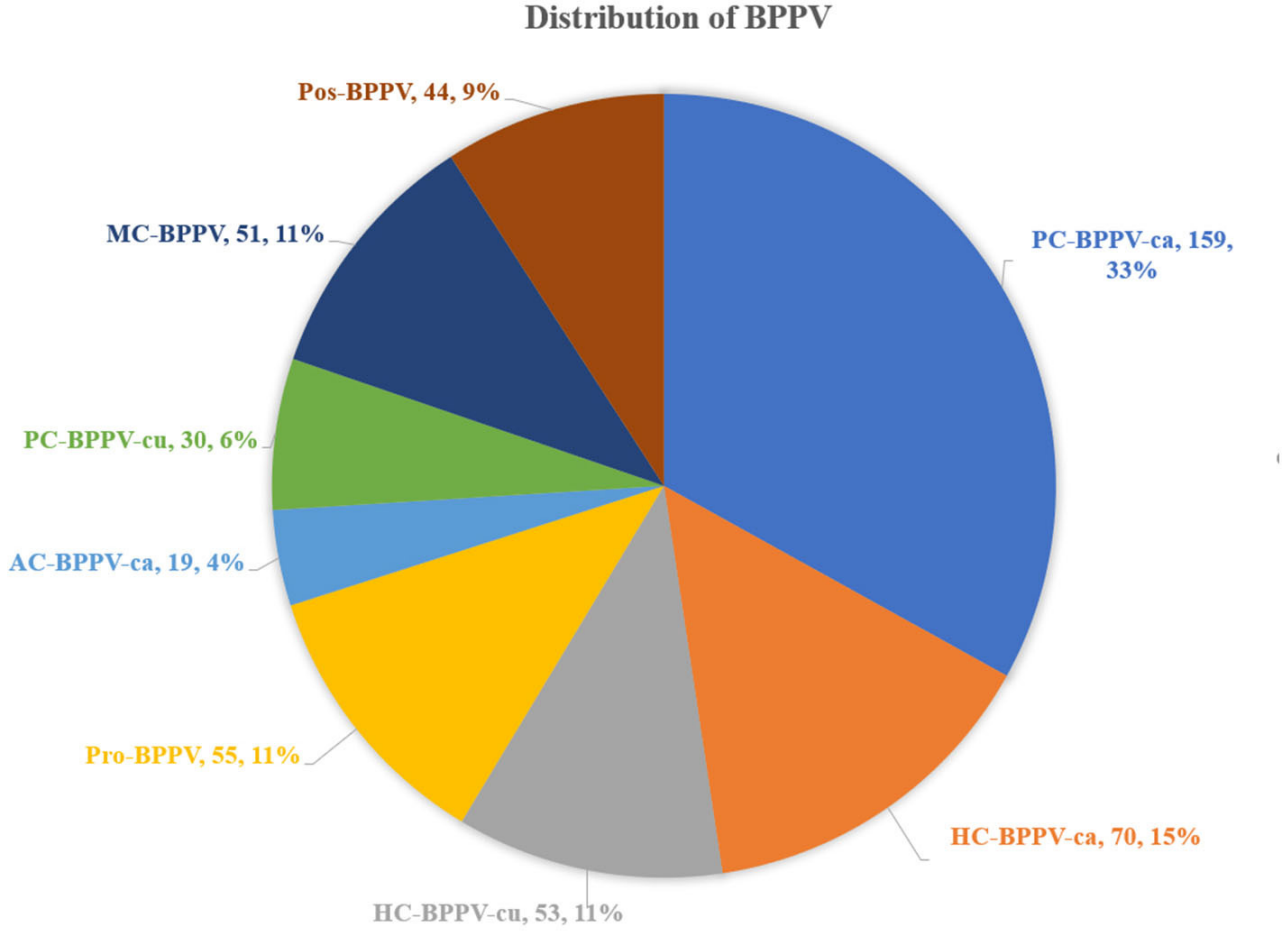
Distribution of all types of BPPV. AC, anterior canal, ca, canalolithiasis, cu, cupulolithiasis; BPPV, benign paroxysmal positional vertigo, HC, horizontal canal, MC, multiple canal, PC, posterior canal, Pro, probably spontaneously resolved, Pos, possible.

The distribution of different types of BPPV: (1) 159 (33.1%) patients had PC-BPPV-ca, 70 (14.6%) patients had HC-BPPV-ca, 55 (11.4%) patients had Pro-BPPV, and 53 (11.0%) patients had HC-BPPV-cu. (2) in emerging and controversial BPPV, 51 (10.6%) patients had MC-BPPV, 44 (9.1%) patients had Pos-BPPV, 30 (6.2%) patients had PC-BPPV-cu and 19 (4.0%) patients had AC-BPPV-ca (Figure 2).

Among the 44 patients with Pos-BPPV, 23 (52.3%) patients showed vertigo without nystagmus induced by the position test, including 17 patients of Pos-PC-BPPV and six patients of Pos-HC-BPPV. Vertigo disappeared in 12 patients following the first particle repositioning therapy. After 1-week and 1-month of treatment, vertigo disappeared in 18 and 21 patients, respectively.

Five (11.4%) patients of the 44 patients with Pos-BPPV were suspected of having multiple canal involvement, but the affected canals were not determined. 4 Patients showed symptoms and nystagmus did not improve after the first particle repositioning therapy, and patients were instructed to perform Brandt-Daroff exercises at home. Vertigo and nystagmus disappeared in 3 patients after one week of follow-up. And after one month of follow-up, vertigo disappeared in 4 patients, recurrence was seen in one patient.

Four (9.1%) patients of the 44 patients with Pos-BPPV had bilateral geotropic positional nystagmus lasting > 1 min when lying on both sides. The slow phase velocity (SPV) of the induced nystagmus was mostly 2–6°/s. Vertigo and nystagmus disappeared in 3 patients after first particle repositioning therapy, which disappeared in all patients after one week of treatment.

4 (9.1%) patients showed apogeotropic nystagmus on the supine roll test when lying on one side, and no nystagmus was observed when lying on the contralateral side. Vertigo disappeared in all patients after first particle repositioning therapy, and vertigo was not recurrent after one week and one month of follow-up. Those patients were diagnosed with possible short-arm HC-BPPV.

Four (9.1%) patients of the 44 patients with Pos-BPPV showed geotropic nystagmus on the supine roll test when lying on one side, and nystagmus was not observed when lying on the contralateral side. Nystagmus disappeared after particle repositioning therapy, and those patients were considered to have possible HC-BPPV.

Three (6.8%) patients had down-beating nystagmus, lasing > 1 min, the direction of the nystagmus was reversed when sitting up. After treatment with Yacovino maneuver, nystagmus was significantly weakened or disappeared, and those patients were considered to have possible AC-BPPV-cu.

One (2.3%) patient of the 44 patients with Pos-BPPV showed transient apogeotropic positional nystagmus on both sides during the supine roll test. The first particle repositioning therapy was ineffective. During one week of follow up, the supine roll test induced geotropic nystagmus which was then converted into apogeotropic nystagmus when lying on the left side, and geotropic nystagmus was induced when lying on the right side. Nystagmus was significantly weakened after treatment with Barbecue maneuver on the left side, and the patient was diagnosed with possible anterior arm HC BPPV (Table 1).

**Table 1:** Clinical features of the Possible BPPV.

**No**	**Sex**	**Age**	**Left DH**	**Right DH**	**Left Roll**	**Right Roll**	**Diagnose**	**Treatment**	**Result**	**One week**	**One month**
1	F	78	CCW+U:35°/s, 15 s → D:9°/s, 50 s	R:7°/s, > 1 min	R:5°/s + D:9°/s,45 s	–	pos–MC-BPPV	Epley+B-D	Effective	Effective	Cure
2	F	71	CCW+U:24°/s, 20 s → D:10°/s, > 1 min	CW+U: 2°/s,20 s → D:10°/s, >1 min	–	–	pos-MC-BPPV	Epley	Cure	Cure	Cure
3	M	27	D:8°/s, 20 s	D:8°/s, 10 s	L:13°/s, 10 s	R:49°/s, 15 s → L:5°/s + D:11°/s, 25 s	pos-MC-BPPV	Yacovino+Barbecue +B-D	Effective	Cure	Cure
4	F	64	U:14°/s > 1 min	CW+U,10°/s, >1 min	L:9°/s, >1 min	R:51°/s, 10s → L:11°/s >1 min	pos-MC-BPPV	Barbecue+B-D	Effective	Cure	Cure
5	M	70	L:7°/s, > 1 min	CW+U:12°/s, 5s → R:6°/s, 38 s	L:6°/s, 35 s	R:5°/s, 30 s	pos-MC-BPPV	Epley+B-D	Effective	Effective	Recurrence
6	M	52	–	–	L:3°/s,>1 min	R:4°/s, > 1 min	Pos-RHC-BPPV	Barbecue	Cure	Cure	Cure
7	M	63	–	–	L:4°/s,>1 min	R:6°/s, > 1 min	Pos-RHC-BPPV	Barbecue	Effective	Cure	Cure
8	F	37	L:4°/s, > 1 min	–	L:3°/s,>1 min	R:2°/s, > 1 min	Pos-LHC-BPPV	Barbecue	Cure	Cure	Cure
9	M	60	L:6°/s, > 1 min	–	L:6°/s,>1 min	R:4°/s, 10s	Pos-LHC-BPPV	Barbecue	Cure	Cure	Cure
10	F	60	–	R:13°/s, 40 s	L:26°/s, 20 s	–	Pos-LHC-BPPV	Barbecue	Cure	Recurrence:RP-RH-BPPV	Cure
11	M	24	–	–	–	R: 7°/s, > 1 min	Pos-RHC-BPPV	Barbecue	Cure	Cure	Cure
12	M	36	–	–	–	R:4°/s, 40s	Pos-RHC-BPPV	Barbecue	Cure	Cure	Cure
13	F	50	–	–	–	R:6°/s, 40s	Pos-RHC-BPPV	Barbecue	Cure	Cure	Cure
14	F	56	–	–	–	L:5°/s, > 1 min	Pos-short arm RHC-BPPV	Barbecue	Cure	Cure	Cure
15	M	46	–	–	–	L:5°/s, > 1 min	Pos-short arm RHC-BPPV	Barbecue	Cure	Cure	Cure
16	M	67	–	–	–	L:6°/s,>1 min	Pos-short arm RHC-BPPV	Barbecue	Cure	Cure	Cure
17	M	46	–	–	R:6°/s, >1 min	–	Pos-short arm LHC-BPPV	Barbecue	Cure	Cure	Cure
18	M	77	–	–	R:41°/s, 30 s	L:23°/s, 28 s	Pos-RHC-BPPV	Head-shaking +Barbecue	Non-effective	Cure	Cure
19	F	41	D:9°/s +R:11°/s,>1 min	–	D:4°/s +R:6°/s,>1 min	–	Pos-AC-BPPV-CU	Yacovino	Cure	Cure	Cure
20	F	62	CCW+D:5°/s, >1 min	CCW+D:4°/s, >1 min	–	–	Pos-AC-BPPV-CU	Yacovino	Cure	Cure	Cure
21	F	63	CW+D:10°/s, >1 min	CW+D: 4°/s, >1 min	–	–	Pos-AC-BPPV-CU	Yacovino	Effective	Cure	Cure
22	F	50	–	VWN	–	–	pos-PC-BPPV	Epley	Cure	Cure	Cure
23	F	59	–	VWN	–	-	pos-PC-BPPV	Epley	Effective	Effective	Cure
24	M	28	–	VWN	–	–	pos-PC-BPPV	Epley	Cure	Cure	Cure
25	F	54	–	VWN	–	–	pos-PC-BPPV	Epley	Cure	Cure	Cure
26	F	36	–	VWN	–	–	pos-PC-BPPV	Epley	Cure	Cure	Cure
27	F	65	–	VWN	–	–	pos-PC-BPPV	Epley	Cure	Cure	Cure
28	F	45	-	VWN	–	–	pos-PC-BPPV	Epley	Cure	Cure	Cure
29	F	64	–	VWN	–	–	pos-PC-BPPV	Epley	Non-effective	Effective	Effective
30	F	54	–	VWN	–	–	pos-PC-BPPV	Epley	Cure	Cure	Cure
31	F	61	–	VWN	–	–	pos-PC-BPPV	Epley	Cure	Cure	Cure
32	F	49	VWN	VWN	–	–	pos-PC-BPPV	Epley	Cure	Cure	Cure
33	F	65	VWN	–	–	–	pos-PC-BPPV	Epley	Effective	Effective	Cure
34	F	69	VWN	–	–	–	pos-PC-BPPV	Epley	Effective	Effective	Cure
35	F	63	VWN	–	–	–	pos-PC-BPPV	Epley	Cure	Cure	Cure
36	F	39	VWN	–	–	–	pos-PC-BPPV	Epley	Effective	Cure	Cure
37	F	71	VWN	–	–	–	pos-PC-BPPV	Epley	Effective	Cure	Cure
38	F	43	VWN	–	–	–	pos-PC-BPPV	Epley	Effective	Cure	Cure
39	F	64	–	–	VWN (much severe)	VWN	pos-HC-BPPV	Barbecue	Effective	Cure	Cure
40	F	78	–	–	VWN (much severe)	VWN	pos-HC-BPPV	Barbecue	Non-effective	Effective	Effective
41	F	30	–	-	VWN	VWN (much severe)	pos-HC-BPPV	Barbecues	Cure	Cure	Cure
42	F	62	–	–	VWN	VWN (much severe)	pos-HC-BPPV	Barbecues	Cure	Cure	Cure
43	F	38	–	- -	VWN	VWN	pos-HC-BPPV	Barbecues	Non-effective	Cure	Cure
44	F	56	–	–	VWN	VWN	pos-HC-BPPV	Barbecues	Non-effective	Cure	Cure

*BPPV, benign paroxysmal positional vertigo; B-D, Brandt-Daroff; CW, clockwise; CCW (from the patien's perspective), counter clockwise (from the patien's perspective); D, downbeat; DH, Dix-Hallpike; HC, horizontal canal; L, left; PC, posterior canal; pos, possible; R, right; VWN, vertigo without nystagmus*.

Among the 51 patients with MC-BPPV, 29 (56.9%) had unilateral side involvement (including 23 patients of right-side involvement and six patients of left-side involvement), 13 (25.5%) patients had bilateral involvement, and the affected side was unclear in 9 (17.6 %) patients.

## Discussion

Previous studies have shown that older people and women were more prone to develop BPPV, with peak onset at about 60 years old, and a male to female ratio of 1:2–3 (7, 8). In this study, we found that the average age of onset was 59.6 ± 14.9 years, and the peak age of onset was 51–70 years old, accounting for 52.4% of all BPPV cases. It is speculated that the likelihood of developing various chronic diseases such as hypertension, diabetes, and hyperlipidemia in older people increased with increasing age, which can cause damage to blood vessels and nerves of the inner ear, leading to disorders of the inner ear microcirculation, and utricle damage, then otoconia can easily fall off and cause the occurrence of BPPV (9–12). In this study, the male: female ratio was 1:2.1, which is basically consistent with the findings of a previous study (1). Studies have found that the high incidence of BPPV in middle-aged and older women is related to the decline in estrogen levels. The reduction in estrogen levels may lead to systemic disturbances in calcium metabolism and affect the synthesis and function of otoliths (13). Studies have found that the incidence of BPPV in women receiving hormone replacement therapy for menopausal symptoms was significantly reduced (14).

Bárány Society's diagnostic criteria for BPPV is a diagnostic guideline jointly created by vestibular specialists from many countries. The diagnostic criteria objectively reflect the clinical status of diagnosis and treatment, and provides an in-depth interpretation of different types of BPPV, which has high clinical application value. In the diagnostic criteria, in addition to the common BPPV types, some rare BPPV types are also included, nystagmus and clinical characteristics of these BPPV types are described, that is useful to clinicians in helping them diagnose BPPV. However, at present, the clinical application of diagnostic criteria for BPPV based on the Bárány Society has been rarely studied and verified. In the present study, we found that PC-BPPV-ca (33.1%) is the most common among patients with definite BPPV, followed by HC-BPPV-ca. (14.6%), Pro-BPPV (11.4%), and HC-BPPV-cu (11.0%). And among patients with emerging and controversial BPPV, MC-BPPV (10.6%) is the most common, followed by Pos-BPPV (9.1 %), PC-BPPV-cu (6.2%), and AC-BPPV-ca (4.0%). There were differences between our results and findings from a study conducted by Yao et al. (15). Yao et al. (15) found that among patients with definite BPPV, PC-BPPV-ca (41.9%) was the most common, followed by Pro-BPPV (11.8%), and HC- BPPV-ca (8.98%), and HC-BPPV-cu (1.76%), while Pos-BPPV (33.98%) is the most common among patients with emerging and controversial BPPV, followed by MC-BPPV (1.23%), AC-BPPV-ca (0.35%), and PC-BPPV-cu (0.00%). The differences in the results between our study and the study mentioned above may be related to the differences in the types of patients included.

At present, AC-BPPV is rarely reported. A study had found that the incidence of AC-BPPV is about 3% (1–17.1%) (16). It is speculated that the low prevalence of AC-BPPV may be mainly related to its spatial anatomical location, the anterior canal is located higher, it is difficult for the otoliths detached from utricle to enter into the anterior canal through crus commune. In our study, the incidence of AC-BPPV was 4%; this result is consistent with the above-mentioned study.

PC-BPPV-cu is rarely reported in previous studies. In our study, the incidence of PC-BPPV-cu was found to be 6.2%. A study showed that in patients with PC-BPPV-cu, half-Dix-Hallpike test induced upbeat nystagmus with a torsional component, the nystagmus had no latency period, did not fatigue, and lasted for more than one minute, a reversal of the direction of nystagmus occurred when sitting up (17). A study also suggested that in patients with PC-BPPV-cu, the nystagmus with torsional component can disappear when the head of patients is hanging backward by an appropriate degree during the Dix-Hallpike test. Because in this position, the cupula of the posterior canal is parallel to gravitational force lines, the cupula stops moving, and the nystagmus stops. When performing the Dix-Hallpike test on the healthy side, upbeat nystagmus with torsional component can be induced, and the upper pole of the eyes beats toward the affected ear (18).

A study had shown that the incidence of MC-BPPV was 6.8 to 20% (19), bilateral posterior canal most frequently involved (20–22). In this study, the incidence of MC-BPPV was 10.6%, a combination of ipsilateral PC-BPPV and HC-BPPV was the most common, accounting for 33.3% (17/51) of all case with MC-BPPV, while bilateral PC-BPPV accounted for only 9.8% (5/51) of the cases, our results are consistent with the results of Lopez-Escamez et al. (23) and Shim et al. (24). A previous study suggested that MC-BPPV is mostly associated with damage to both ears caused by traumatic brain injury (25). However, the incidence of traumatic brain injury in MC-BPPV patients was relatively low in our study; only two patients with bilateral PC-BPPV had traumatic brain injury. This may be the reason for the lower incidence of bilateral PC-BPPV. Besides, our research found that MC-BPPV was more likely to be unilateral (29/51, 56.9%); the right side was more commonly affected than the left side. This may be related to the fact that BPPV was more likely to involve the right side (26, 27).

In this study, the incidence of Pro-BPPV was 11.4%, which was basically consistent with the findings of Yao et al. (15), the authors reported a frequency of 11.8%. Studies had shown that the self-healing and atypical symptoms in BPPV patients are associated with their delayed hospital visit (28). The longer the time from onset of symptoms to hospital visits, the higher the incidence of Pro-BPPV. For patients whose symptoms have been completely improved during the visit, it is essential to differentiate BPPV from other paroxysmal vestibular diseases (such as vestibular paroxysmia (29), vestibular migraine (30), and TIA), to avoid misdiagnosis.

At present, a few studies have investigated Pos-BPPV. In our study, the incidence of Pos-BPPV was 9.1%. (1) Among patients with Pos-BPPV, many patients had symptoms of dizziness/vertigo but did not have prominent nystagmus during the position test, and this was called subjective BPPV. Liu et al. (31) found that the incidence of Pos-BPPV was about 13.1% (121/922), particle repositioning therapy was performed according to the symptoms of dizziness/vertigo, and the treatment effect for patients with Pos-BPPV was the same as for patients with definite BPPV. The results of our study are consistent with the above results. The reasons for patients with Pos-BPPV who only had symptoms of dizziness/vertigo and no nystagmus may be as follows: (a) the number of detached otoliths is small, or the otoliths are dispersed in the semicircular canal, which can not cause endolymph flow when moving in the canals, and cannot reach the threshold for inducing vestibular ocular reflex; (b) patients may experience recurrent episodes of vertigo before their visit, and due to the fatigue of nystagmus, patients only showed vertigo and no nystagmus during the position test (32); (c) patients had taken vestibular suppressants (such as benzodiazepine, antihistamine, and anticholinergic drugs) before their visit, which can cause suppression of vestibular function and reduced sensitivity to the position test (33); (d) nystagmus may not be seen with the naked eye when the nystagmus is weak. Therefore, patients with an initial diagnosis of Pos-BPPV should be followed up closely. Some patients may convert to definite BPPV, but some patients may have other vestibular-related diseases. (2) Five patients (11.4%) were suspected of having multiple canal involvement, but the affected canals cannot be determined. It is speculated that the three semicircular canals are arranged orthogonally when the otoliths are present in different semicircular canals and different positions of the canals, the Dix-Hallpike or Roll tests can cause the movement of the otoliths in multiple canals, the nystagmus induced can be superimposed or cancel each other, so the types of nystagmus induced are different, resulting in difficulties in determining the affected canals. For such patients, we first adopted conventional particle repositioning therapy. After treatment, symptoms of vertigo and nystagmus were relieved in 1 patient. However, the nystagmus was slightly reduced in the remaining four patients, but nystagmus still existed, so these patients were instructed to perform the Brandt-Daroff exercises (three times per day) after returning home. After one week, vertigo and nystagmus have disappeared in 3 patients during re-examination after one week, which have disappeared in all four patients after one month, recurrence was observed in one patient. Amor-Dorado et al. (34) compared the therapeutic efficacy of Brandt-Daroff exercise and particle repositioning maneuvers in PC-BPPV patients, and found that its short-term efficacy of Brandt-Daroff exercise is not better than particle repositioning maneuvers. Therefore, particle repositioning maneuvers can be the first choice for patients whose involved semicircular canal is determined. Still, for patients whose involved semicircular canal and affected side are difficult to determine, Brandt-Daroff exercises can be used as an alternative treatment. (3) Four patients (9.8%) had geotropic paroxysmal nystagmus lasting > 1 min when lying on both sides. The slow phase velocity (SPV) was 2–6°/s. It is speculated that due to the anatomical position of the horizontal canal or the small number of otolith particles in the canals, the otoconia move slowly under the influence of gravity during the supine roll test, it will take a long time to move from the position of the canal in supine position to the lowest position of the canal in a lateral position, so the intensity of the nystagmus induced was weaker and has a long duration. (4) Four (9.1%) patients with Pos-BPPV showed apogeotropic nystagmus on roll test when lying on one side, and no nystagmus was observed when lying on the other side, those patients were diagnosed with possible short arm HC-BPPV (35). One of the patients had a history of PC-BPPV-ca half a month ago. It is speculated that when the otoliths are located in the short arm of the semicircular canal, the otoliths move from the short arm to cupula if the patients lie on their affected side, cupula moves away from the ampulla, apogeotropic nystagmus was induced. When the patients lie on their healthy side, the otoliths move away from the ampulla and fall into the utricle, so no nystagmus was induced (35). (5) Four (9.1%) patients showed geotropic nystagmus on the supine roll test when lying on one side, and nystagmus was not observed when lying on the contralateral side. Nystagmus disappeared after particle repositioning therapy, and those patients were considered to have possible HC-BPPV. In 3 of these 4 patients, the geotropic nystagmus is weak, the SPV was about 4–7°/s. It is speculated that with spontaneous movement of patients, most of the otoliths may be restored themselves. At this time, there are few otolith particles in the semicircular canal, so geotropic nystagmus was only induced on one side. In the remaining one patient, left-beating geotropic nystagmus was only induced when lying on the left side during the supine roll test, and right-beating geotropic nystagmus was induced during right Dix-Hallpike test, the nystagmus disappeared after particle repositioning therapy, which may be related to the uncertainty of the movement of otoliths. (6) In the present study, three patients showed downbeat nystagmus lasting > 1 min during Dix-Hallpike, the nystagmus had no latency period and did not fatigue, and the reversal of the nystagmus occurred when sitting up, those patients had a history of transient episodes of vertigo triggered by changes in body posture. Yacovino maneuver was effective in those patients; the nystagmus disappeared or was significantly weakened after treatment, therefore, those patients were diagnosed as possible AC-BPPV-cu (6). At present, there are relatively few studies investigating AC-BPPV-cu, and there is still controversy about the existence of AC-BPPV-cu. Adamec and Habek (36) reported a case of AC-BPPV-cu, this patient had a previous history of PC-BPPV, and the nystagmus completely disappeared after treatment with Yacovino maneuver. Dlugaiczy et al. (37) suggested that compared with canalolithiasis, cupulolithiasis may be a more common type of AC-BPPV caused by brain trauma. It should be noted that positional downbeat nystagmus is more commonly seen in the vestibulocerebellum, craniocerebral junction injuries or drug poisoning (38). Therefore, during the diagnosis of AC-BPPV-cu, the above diseases should be considered in the differential diagnosis to avoid misdiagnosis. (7) One (2.4%) patient showed transient apogeotropic positional nystagmus on both sides during the supine roll test. The first particle repositioning therapy was ineffective. During one week of follow up, the supine roll test induced stronger geotropic nystagmus which was then converted into apogeotropic nystagmus when lying on the left side, and the geotropic nystagmus was induced when lying on the right side. Nystagmus was significantly weakened after treatment with Barbecue maneuver on the left side, and the patient was diagnosed with possible anterior arm HC BPPV. As can be seen from above, during the diagnosis of Pos-BPPV, the history of typical BPPV, particle repositioning therapy and dynamic follow-up outcome contribute to accurate diagnosis of Pos-BPPV.

## Limitations

Our study is a single-center research, our study does not represent the general population, and the sample size is small. Further multi-center studies with a larger sample are needed to confirm the findings.

## Conclusion

Among patients with BPPV, PC-BPPV-ca is most common, followed by HC-BPPV-ca. Among patients with emerging and controversial BPPV, MC-BPPV, and Pos-BPPV were more common. For the diagnosis of Pos-BPPV, a combination of the history of typical BPPV, particle repositioning therapy, and follow-up outcome is helpful to clarify the diagnosis.

## Data Availability Statement

The datasets generated for this study are available on request to the corresponding author.

## Ethics Statement

The studies involving human participants were reviewed and approved by Aerospace Center Hospital, Peking University Aerospace School of Clinical Medicine. The patients/participants provided their written informed consent to participate in this study.

## Author Contributions

XY contributed to the conception and design of the study. XL and D-HZ collected the clinical data. BS, L-HS, K-ZL, YH, and Z-YL analyzed the results. XL, D-HZ, and XY drafted and corrected the manuscript. All authors contributed to the article and approved the submitted version.

## Conflict of Interest

The authors declare that the research was conducted in the absence of any commercial or financial relationships that could be construed as a potential conflict of interest. The handling editor declared a past co-authorship with one of the authors XY.
